# Bezafibrate-driven mitochondrial targeting enhances antitumor immunity and prevents lung cancer via CD8+ T cell infiltration and MDSC reduction

**DOI:** 10.3389/fimmu.2025.1539808

**Published:** 2025-04-15

**Authors:** Jing Pan, Jiaheng Li, Qi Zhang, Mofei Huang, Yian Wang, Ming You

**Affiliations:** Center for Cancer Prevention, Dr. Mary and Ron Neal Cancer Center, Houston Methodist Research Institute, Houston, TX, United States

**Keywords:** Bezafibrate, mitochondria-targeted compound, tumor immune microenvironment, CD8, complex III

## Abstract

Bezafibrate (BEZ) is a drug used to treat hypertriglyceridemia and its long-term use has been associated with reduced risk of cancer in patients with coronary artery disease. Recent studies uncovered that BEZ is a potent modulator of mitochondrial biogenesis through activation of PGC-1α/PPAR complexes, resulting in modulation of lipid metabolism and fatty acid oxidation. Mitochondria impact virtually all processes linked to oncogenesis, and disruption of normal mitochondrial bioenergetics and oxidative phosphorylation (OXPHOS) occurs early during oncogenesis to change the energy metabolism of cancer cells as well as various cells in the tumor microenvironment (TME). Therefore, we synthesized a BEZ analog (Mito-BEZ) that preferentially localizes to mitochondria, thereby enabling lower doses of Mito-BEZ than BEZ to achieve greater efficacy. Our studies demonstrate that Mito-BEZ is significantly more potent than BEZ at inhibiting LUAD cell growth *in vitro* and inhibiting lung tumorigenesis in preclinical mouse models. Mito-BEZ was also >200-fold more potent than BEZ at inhibiting both complex I and III in LUAD cells. Furthermore, Mito-BEZ suppresses oxidative metabolism in cancer cells while markedly upregulating mitochondrial function in effector CD8+ T cells, resulting in activation of a potent T cell immune response in the TME. Our results show that Mito-BEZ, with its favorable toxicity profile, exhibited a striking inhibitory effect on lung cancer progression and metastasis by targeting a fundamental difference in metabolic plasticity between cancer cells and effector T cells in the TME.

## Introduction

Non-small-cell lung cancers (NSCLCs) are the most common lung cancers, accounting for >80% of all lung cancer cases in the United States. Cigarette smoking is the predominant cause of this disease and former smokers remain at elevated risk. About 40% of NSCLCs consist of lung adenocarcinomas (LUAD). Chemoprevention of LUAD development in at-risk populations such as former smokers is an attractive strategy to reduce mortality in NSCLC patients, but additional strategies to prevent primary and metastatic LUAD are needed.

Mitochondria can influence all key processes linked to early oncogenesis. Alteration of normal mitochondrial bioenergetics and oxidative phosphorylation (OXPHOS) occurs early during oncogenesis, which changes the energy metabolism of cancer cells as well as various cells in the tumor microenvironment (TME). We recently demonstrated that novel OXPHOS inhibitors not only prevent tumor growth and metastasis by directly targeting cancer cell mitochondria but also trigger potent T cell immune responses by reversing immunosuppression in the TME (1–3).

Bezafibrate (BEZ) has been used to reduce LDL and triglyceride levels and is a known PPAR agonist that promotes transcription of many genes involved in fatty acid oxidation and apolipoprotein production and has been studied extensively for mitochondria disorders (4). BEZ treatment is associated with reduced risk of cancer in epidemiology studies and has shown specific inhibitory effects on both primary and metastatic lung cancer growth in rodent Kras^G12D^ mutation models (5). PPARγ is upregulated in human lung cancer and activation of PPARγ inhibits lung cancer cell growth (6). PPARγ activating drugs are associated with a lower risk of developing lung cancer and have been shown to prevent smoke-induced lung cancer in mice. The beneficial effects of PPARα ligands have also been recently discovered mainly due to their ability to promote the generation of anti-angiogenic factors (7) or to decrease endothelial Cyp2c44 expression and EET biosynthesis (5). BEZ has been shown to inhibit tumorigenesis (8), inflammation (9–11), and invasion and metastasis (5) by activating multiple PPARs including PPARγ and PPARα, subsequently resulting in the modulation of multiple cell growth and survival-related signaling pathways in cancer cells. BEZ also modulates mitochondrial and redox pathways (12, 13).

Our previous studies showed that agents targeting cancer cell bioenergetics are promising for inhibiting tumor growth and are less toxic to normal cells (2, 3). Increased negative plasma membrane and mitochondrial transmembrane potentials in cancer cells facilitate the selective accumulation and retention of delocalized lipophilic cations such as rhodamine 123 (14–16). Several mitochondria-targeted agents containing a triphenylphosphonium ion (TPP^+^) attached to a bioactive molecule (e.g., Mito-Q) decrease ATP levels more potently in cancer cells compared to normal cells, and inhibit cancer cell proliferation at nontoxic sub-micromolar levels (2, 3). Although tumor cells rely to a large extent on aerobic glycolysis to generate ATP (the Warburg effect), mitochondria are indeed functional in most tumor cells (17–20). Detailed profiling of cellular bioenergetics has recently provided new insights into the intermediacy of mitochondrial metabolism in tumor cells (18, 21). Thus, targeting mitochondrial bioenergetics is emerging as an effective and viable preventive approach to inhibit the proliferation of tumor cells (2, 3, 17, 20).

In this study, we synthesized a novel mitochondria‐targeted BEZ (Mito-BEZ) compound by attaching the bulky TPP^+^ group to BEZ via a long alkyl chain, which separates TPP^+^ from BEZ and increases its lipophilicity and mitochondrial uptake in cells. Mito-BEZ is over 100-fold more potent in inhibiting mitochondrial oxygen consumption than BEZ. Mito-BEZ also improved the antitumor activity of PD-1-blocking immunotherapy. We found that Mito-BEZ treatment increased CD8^+^ T cells and decreased MDSC. Our results show that Mito-BEZ exhibited a more striking inhibitory effect than BEZ on lung cancer progression by targeting metabolic plasticity in effector T cells in the TME. Mito-BEZ is thus a novel, potent, chemopreventive agent of lung tumor progression and metastasis that acts primarily through mitochondrial mechanisms.

## Methods

### Synthesis of Mito-BEZ

Mito-Bezafibrate (Mito-BEZ) was synthesized by attaching TPP^+^ (triphenylphosphonium cation) via amidation between BEZ and an aminoalkyl-TPP^+^ linker. The products were purified by silica gel chromatography, and their structures were characterized by NMR (**Supplementary Materials**, **Supplementary Figures S1–3**).

### Cell lines and animals

LKR13 cells were a generous gift from Dr. Jonathan M. Kurie (MD Anderson). LKR13‐luc cells were generated by transfecting LKR13 cells with CMV‐firefly luciferase lentivirus (Cellomics Technology). LLC cells and CT26 cells were purchased from ATCC. H2030BrM3 and PC9BrM3 cells were generously provided by Dr. Joan Massagué (Cancer Biology and Genetics Program, Memorial Sloan Kettering Cancer Center, New York, NY). Normal lung cell line NHBE, was obtained from Lonza. B16 and B16 p0 cells were generously provided by Dr. Martina Bajzikova (Institute of Biotechnology, Czech Academy of Sciences, Czech Republic). LKR-13, CT26, H2030BrM3 and PC9BrM3 were cultured in a complete medium consisting of RPMI‐1640 (Thermofisher) supplemented with 10% fetal bovine serum (Sigma) and 1% penicillin/streptomycin (Gibco). LLC cells were cultured in DMEM (Gibco) supplemented with 10% FBS, and 1% penicillin/streptomycin (Gibco). B16 and B16 p0 were cultured in DMEM (Gibco) supplemented with 10% FBS, 1% penicillin/streptomycin (Gibco), sodium pyruvate (1 mM), and uridine (50 µg/ml). NHBE cells were cultured in BEGM bronchial epithelial cell growth medium (Lonza). All cell lines used in this study were authenticated and verified to be free of *Mycoplasma* contamination (Universal Mycoplasma Detection Kit, ATCC). Vinyl carbamate was purchased from Santa Cruz.

Female SV129 and female C57BL/6 mice were purchased from the Jackson Laboratory. SMARTA triple reporter mice were generated by crossbreeding SMARTA mice (Jackson Laboratory, Stock No. 030450) with GREAT mice (interferon-gamma reporter with endogenous polyA transcript, Jackson Laboratory, Stock No. 017581) and Foxp3^EGFP^ mice (co-express EGFP and the regulatory T cell-specific transcription factor Foxp3, Jackson Laboratory, Stock No. 006772). All studies on animals were approved by the Houston Methodist Research Institute Institutional Animal Care and Use Committee (approval number: IS00006363).

### Cellular proliferation assay

LKR-13 (2 × 10^3^ cells/well) and LLC (2 × 10^3^ cells/well) were plated in 96-well plates, treated with Mito-BEZ or vehicle control (DMSO), and cell proliferation was measured using a label-free, noninvasive cellular confluence assay (IncuCyte Live Cell Imaging Systems, IncuCyte FLR, Essen Bioscience, Ann Arbor, MI) as recommended by the manufacturer.

### Mitochondrial function in intact and permeabilized cells

Mitochondrial function was monitored using a Seahorse XF96 Extracellular Flux Analyzer (Agilent, Santa Clara, CA) (17). For cancer cells, 2×10^5^ cells were seeded in specialized V7 Seahorse tissue culture 96-well plates 24 hours before the analysis and maintained at 37°C in 5% CO_2_. After seeding and treatment as indicated, cells were washed with unbuffered media as described above. Three baseline OCR and ECAR measurements were then taken before injection of oligomycin (1 μg/mL) to inhibit ATP synthase, carbonylcyanide p-(trifluoromethoxy) phenylhydrazone (FCCP; 1–3 μmol/L) to uncouple the mitochondria and yield maximal OCR, and antimycin A (10 μmol/L) to prevent mitochondrial oxygen consumption through inhibition of complex III. From these measurements, indices of mitochondrial function were determined as manufacture described. For CD8+ T cells, naïve CD8+ T cells were first negatively isolated from the spleens of the naïve Friend Virus B Type (FVB) mice using a separation cocktail (104-075, Miltenyi Biotec, Bergisch Gladbach, North Rhine-Westphalia, Germany) and large scale (LS) column (130-042-401, Miltenyi Biotec). Naïve CD8+ T cells were plated in 12-well plate (2 × 10^6^ cells/well), activated with plate-bound anti-CD3 (1 µg/mL, 100238, Biolegend), soluble anti-CD28 (2 µg/mL, 102116, Biolegend) and mouse IL-2 (100 U/mL, 14-0821-64, Thermo Fisher) in the presence of vehicle or Mito-BEZ (0.025 ~ 0.25 μmol/L) for 2~3 days. Cells were then harvested for seahorse analysis. For oxygen consumption rate (OCR) and extracellular acidification rate (ECAR) determination, 2×10^5^ cells were plated on poly-D-lysine (50 μg/mL, A3890401, Thermo Fisher) coated seahorse cell plate and were incubated in XF Base Medium (Seahorse Bioscience) supplemented with 2 mmol/L glutamine, 10 mmol/L glucose, and 1 mmol/L pyruvate for 1 h in a non-CO2 incubator. CD8+ T Cells were analyzed under stressed conditions and in response to oligomycin (1.5 μmol/L), carbonyl cyanide 4-(trifluoromethoxy) phenylhydrazone (FCCP; 1.5 μmol/L), rotenone and antimycin A (R/A, 1 μmol/L) using the Seahorse XF cell Mito Stress kit (103015-100, Agilent Technologies, Santa Clara, CA, USA).

Mitochondrial respiratory complex activity in permeabilized cells was measured according to the manufacturer’s instructions. Briefly, intact cells were permeabilized using 1 nmol/L Plasma Membrane Permeabilizer (PMP, Agilent) immediately before measuring the oxygen consumption rate (OCR). OCR derived from mitochondrial complexes was measured using different mitochondrial substrates (e.g., pyruvate/malate for complex I and succinate for complex II) with a Seahorse XF96 Extracellular Flux Analyzer (Agilent, Santa Clara, CA). Rotenone, malonate, and antimycin A were used as specific inhibitors of mitochondrial complexes I, II, and III, respectively. All OCR and ECAR reports were generated by Wave Desktop software (Agilent Technologies).

### Combination of Mito-BEZ treatment and anti-PD-1 treatment

Six-week-old female C57BL/6 or BALB/c mice were purchased from the Jackson Laboratory and inoculated with LLC tumor cells or CT26 cells separately. Animals were randomized into different treatment groups: (a) vehicle control, (b) anti-PD-1 (Bioxcell, BE0146) (200 µg/mouse, i.p. every other day) (c) Mito-BEZ (3 µmol/kg·bw, oral gavage daily), (d) combination. Mice started treatments on day 7 after tumor inoculation and tumor sizes were measured every two days. Mice were followed until death or euthanized earlier if tumors reached 4000 mm^3^.

### *Ex vivo* T cell activation assay using SMARTA triple reporter mice

To differentiate CD4+ T cells into a T regulatory cell phenotype, splenocytes from SMARTA triple-reporter mice were processed, and the red blood cell was lysed using an ACK (ammonium-chloride-potassium) lysis buffer. The cells were then activated with 1 μg/mL GP61–80 peptide (GenScript, Piscataway, NJ), 5 ng/mL TGF-β1 (Shenandoah Biotechnology, Inc., Warwick, PA), and 1 μg/mL CD28 in 24-well plates precoated with CD3 (2.5ug/ml). After one day of initial skewing, 100 U/mL IL-2 along with BEZ or Mito-BEZ analogs of varying concentrations were added to the culture. Cells were split on day 2 and day 4 with IL-2 and BEZ or Mito-BEZ replenished at the same concentration. After six days in culture, cells were blocked with BFA/Monensin for 4 hours, and then stained to assess the viability and phenotypic analysis via flow cytometry. LIVE/DEAD fixable violet dead cell stain (Invitrogen, Carlsbad, CA) was used to assess cell viability. The following antibodies were used for flow cytometry staining: PercP anti-mouse CD45 (clone: 30-F11), APC/Cy7 anti-mouse CD25 (clone: PC61), APC anti-mouse FOXP3 (clone: FJK-16S), BV510 anti-mouse CD8 (Clone: 53-6.7), PE-Cy7 anti-mouse CD4 (Clone: GK1.5) and PE anti-mouse IFN*-γ*. Flow cytometry data were acquired using a BD Fortessa X-20 (BD Biosciences, CA) flow cytometer and analyzed using FlowJo (Treestar, Inc., Ashland, OR).

### Flow cytometry

Tumors were minced into 2 mm^3^ pieces and digested with mouse tumor dissociation buffer (Miltenyi Biotec, CA, 130-096-730) at 37°C for 30 min and passed through a 40-µm nylon mesh to generate single-cell suspensions per the manufacturer’s instructions. Red blood cells were removed by red blood cell lysis buffer (1.55 mM NH_4_Cl, 1 mM KHCO_3_, 0.1 mM EDTA). Isolated cells were first stained for viability and cell surface markers. Violet fluorescent reactive dye (Invitrogen, MP34955) was used to identify viable cells. Antibodies for staining surface markers included: BV786 anti-CD45 (Clone: 30-F11), PE anti-CD3 (Clone: 17A2), FITC anti-CD4 (Clone: GK1.5), BUV396 anti-CD8a (Clone: 53-6.7), FITC anti-CD11b (Clone: M1/70), APC/Fire750 anti-F4/80 (Clone: BM8), BUV396 anti-Ly6G (Clone: 1A8), PE/Cy7 anti- Ly6C (Clone: HK1.4), and APC/Fire750 anti-CD25 (Clone: PC61). For transcription factor staining, cells were first stained with surface markers, then fixed with fixation buffer (Biolegend, 420801), permeabilized with FoxP3/Transcription Factor Staining Buffer Set (eBioscience, 00-5523-00), and stained with APC anti-FoxP3 (Clone: FJK-16s). For intracellular cytokine staining, cells were stimulated for 4 h at 37°C in Roswell Park Memorial Institute medium containing 10% fetal bovine serum, 2 × mM l-glutamine, 50 µM 2-mercaptoethanol, 1% penicillin-streptomycin, 0.2% cell stimulation cocktail (eBioscience, 00-4970-93), 0.1% monensin (eBioscience, 00-4505-51), and Brefeldin A (eBioscience, 00-4506-51). Cells were surface-stained with antibodies, fixed, and permeabilized using FoxP3/transcription factor staining buffer set, stained with intracellular cytokine staining buffer containing PE anti-IFN-*γ* and PE-Cy7 anti-TNF-α antibodies, and finally, analyzed by flow cytometry. Cells incubated in a medium lacking PMA/ionomycin served as nonstimulated controls. To analyze myeloid-derived cells, cells were additionally incubated with anti-Mo CD16/CD32 (Invitrogen, 14-0161-82). These flow cytometry antibodies were purchased from either Biolegend, eBioscience, or BD Biosciences. Cells were analyzed using an LSR Fortessa X-20 flow cytometer (Becton Dickinson). Data were analyzed using FlowJo software (Treestar, Inc.).

### Statistical analysis

GraphPad Prism software was used for evaluating statistical differences between treatments. Student’s *t*-test was applied for pairwise comparisons. Multiple comparisons (e.g., inhibition of viability data) were assessed using ANOVA with Tukey’s *post-hoc* test. *P*-Values of < 0.05 were considered significant.

## Results

### Synthesis and the cell proliferation assay of Mito-BEZ

**Figure 1:**
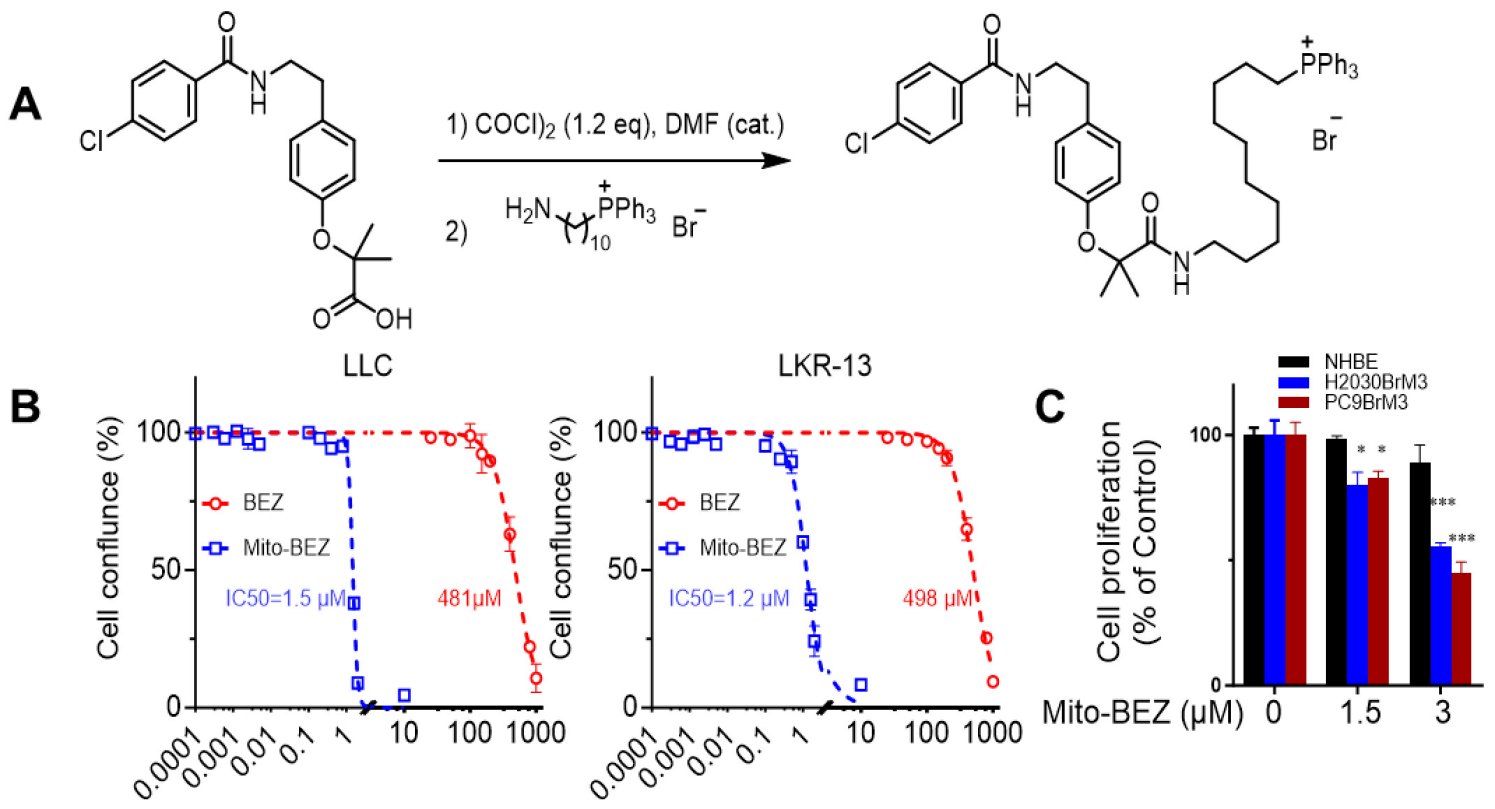
Mito-BEZ has potent anti-proliferative activities on tumor cells. **(A)** Method of Mito-BEZ synthesis. **(B)** Effect of Mito-BEZ on the proliferation of LKR13 and LLC cells. **(C)** Effect of Mito-BEZ on the proliferation of normal human bronchial epithelial NHBE cells. *p ≤ 0.05, ***p ≤ 0.001.

Mito-BEZ links Bezafibrate to TPP+ via a long alkyl chain. The alkyl chain in Mito-BEZ separates the bulky TPP+ group from the core structure of BEZ, minimizing the effect of TPP+ on the pharmacophore. Mito-BEZ was synthesized by converting the parental compound BEZ into an acyl chloride intermediate, followed by a sequential nucleophilic substitution with aminoacyl-TPP+ bromide (**Figure 1A**, **Supplementary Figures S1–3**). The product was isolated from gel chromatography and characterized by NMR.

Antiproliferative effects of Mito-BEZ were compared to BEZ in LKR-13 LUAD and LLC LUAD cells. Using the IncuCyte Live-Cell Imaging Analyzer for real-time cell confluence data, Mito-BEZ inhibits proliferation at significantly lower levels (IC_50_ = 1.5 µM) than BEZ (IC_50_ = 483.1µM) in LLC and LKR13 (Mito-BEZ IC_50c_= 1.5 µM and BEZ IC_50_ = 498 µM) cell lines (**Figure 1B**). As shown in **Figure 1C**, Mito-BEZ also selectively to inhibit cell proliferation in tumorigenic human H2030BrM3 and PC9BrM3 cells, but not in normal human NHBE cells. These data indicate that Mito-BEZ’s induction of cell death occurs specifically in cancer cell lines, with limited effects on normal cells.

### Mito-BEZ inhibits mitochondrial complexes I and III activity

**Figure 2:**
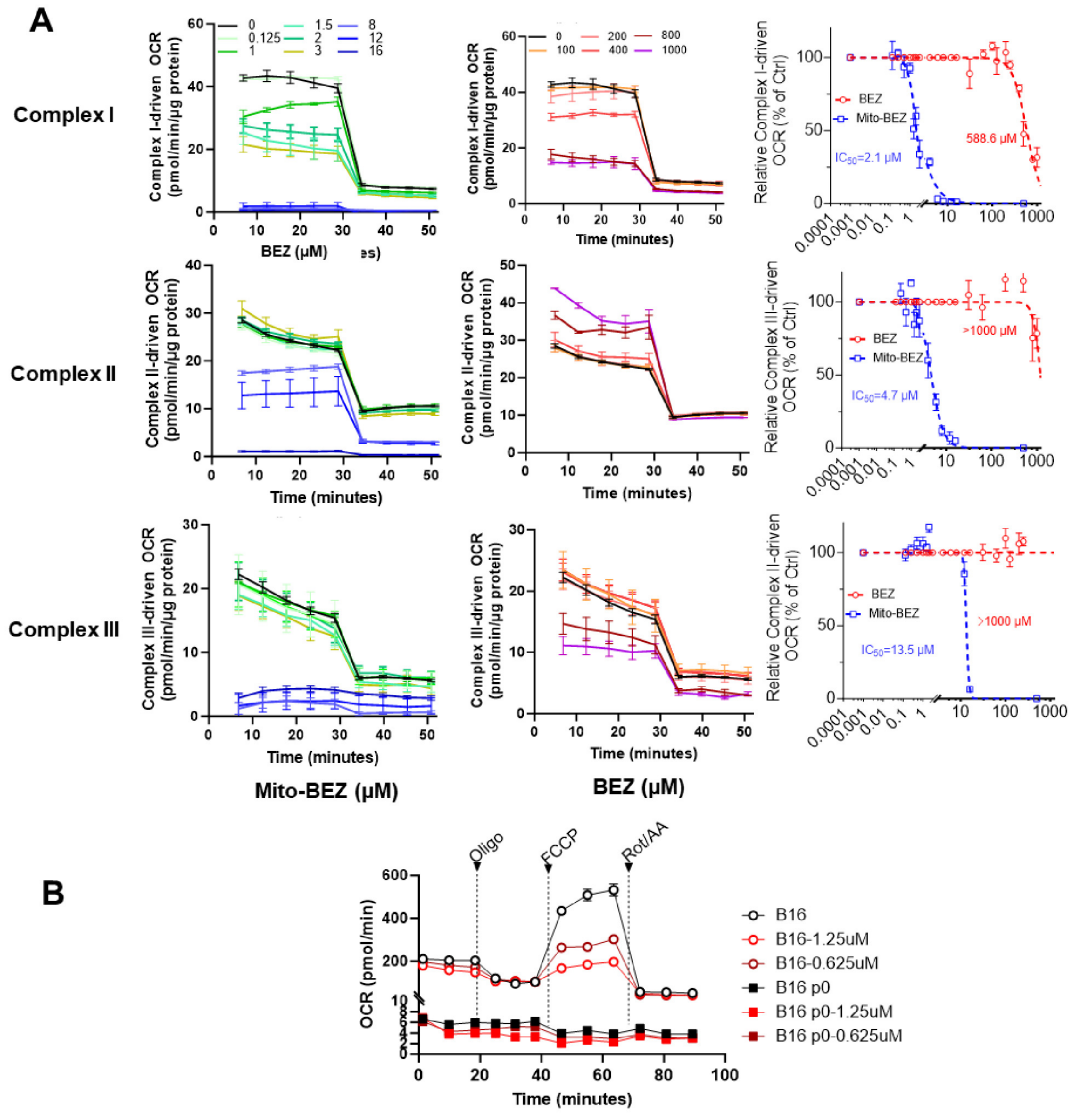
Mito-BEZ is a potent inhibitor of mitochondrial complexes I and III. **(A)** Effects of BEZ and Mito-BEZ on the activity of mitochondrial complexes I, II, and III. Selected Seahorse XF OCR traces for Mito-BEZ (top panels) or BEZ (middle panels), with the injection of inhibitors of complex I (rotenone, ROT), complex II (Malonate), or complex III (antimycin A, AA). Bottom panels: IC50 of the concentration curves for inhibition of complex I, II, or complex III by BEZ and Mito-BEZ. **(B)** Effects of Mito-BEZ on mitochondria-depleted B16 p0 cells compared to its parental B16 cells.

To test the effect of BEZ and Mito-BEZ on mitochondrial complex activity, LKR13 cells were pretreated for 24 h with the compounds, followed by cell membrane permeabilization, the addition of complex substrates/inhibitors, and OCR measurements. Rotenone (complex I inhibitor) was used to confirm complex I activity, while antimycin A was used as a complex III inhibitor (22). We observed that both Mito-BEZ and BEZ could inhibit complex I activity, with the IC50 for Mito-BEZ being 2.1 µM compared to BEZ at 588.6 µM (**Figure 2A** top panels). Mito-BEZ also inhibited complexes II and III, however, compared to Complex II (IC50 = 13.5 µM), Mito-BEZ had significant inhibition with much lower IC50 for Complex III (IC50 = 4.7 µM), while BEZ had no inhibitory activity at doses over 1000 µM (**Figure 2A** middle and bottom panels). These results indicate that the new BEZ analog showed significantly enhanced mitochondria-targeting potential, and could be a very potent inhibitor for both complexes I and III.

To further validate if Mito-BEZ depends on mitochondria for its anti-cancer function, we tested Mito-BEZ in a pair of cancer cell lines with verified deficient mitochondria (23, 24), one parental B16F10 melanoma cells and its mitochondrial-deficient derivative ρ0 cells, which has host mtDNA depleted with a truncated form of a viral UL12.5 gene. Depletion of mtDNA was demonstrated by a lack of mitochondrial complex I and II activities (**Figure 2B**), and Mito-BEZ treatment inhibited mitochondria function at different doses in the parental B16 cell lines but showed only minimal effects on ρ0 cells (**Figure 2B**). These results suggest that the anti-cancer effects of Mito-BEZ are dependent on mitochondrial function.

### Mito-BEZ increases tumor-infiltrating CD8^+^ T cells and reduces MDSCs in tumors

**Figure 3:**
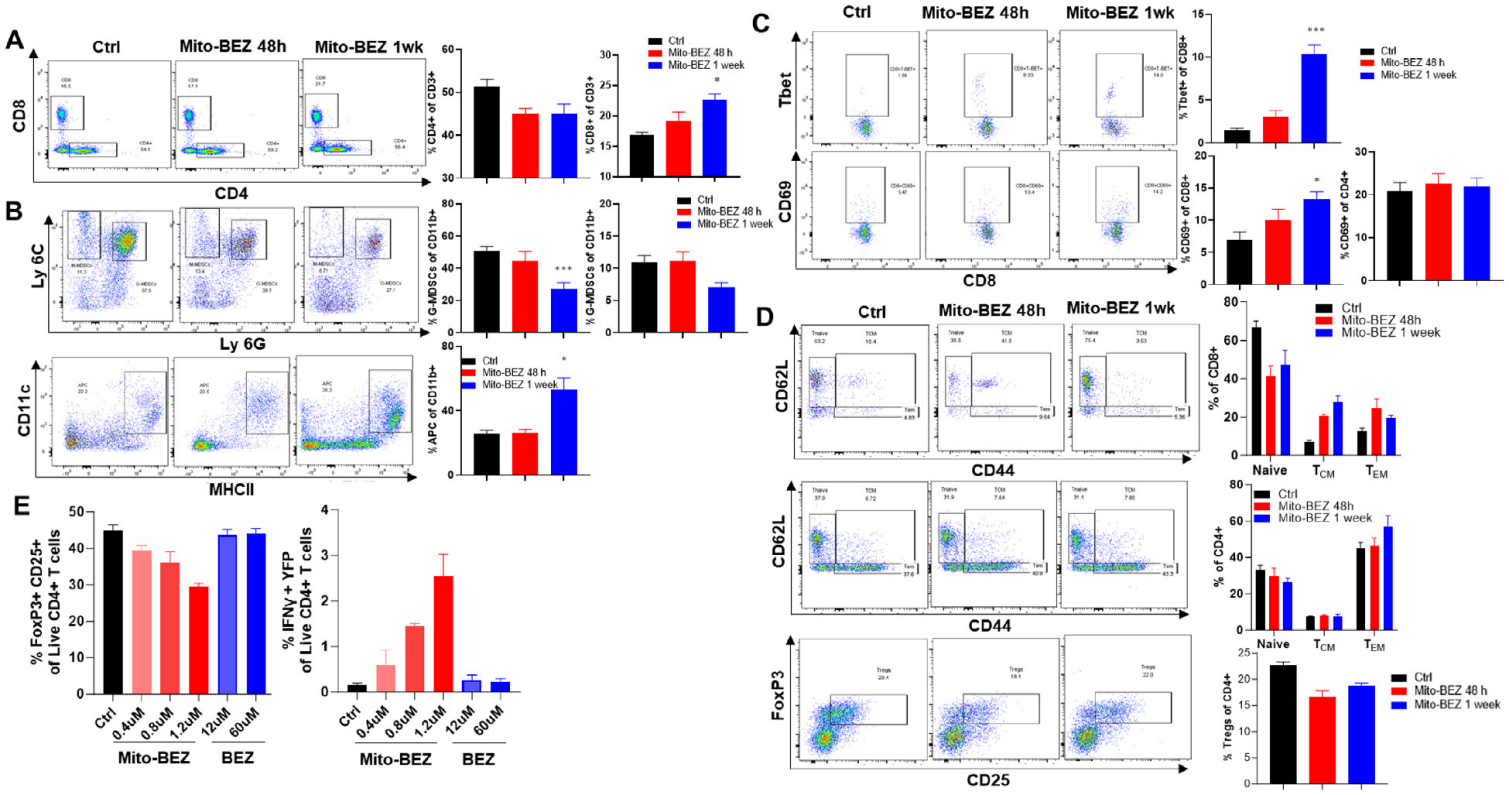
Mito-BEZ increases the presence of tumor-infiltrating CD8^+^ T cells and decreases MDSC cells. **(A)** Percent of CD8 and CD4 T cells. **(B)** G-MDSCs, M-MDSCs, and antigen-presenting cells. **(C)** Tbet+ and CD69+ T cells. **(D)** Effector memory T cells and Treg cells. **(E)** *In vitro* Treg cell function assay in SMARTA mice. *p ≤ 0.05, **p ≤ 0.01, ***p ≤ 0.001.

To evaluate whether Mito-BEZ modulates the tumor immune microenvironment, we conducted multicolor flow cytometric analyses from the TME of mice implanted with LKR13 cells that were treated with 3 μmol/kg·bw of Mito-BEZ by oral gavage at different time points. Mito-BEZ treatment led to an increased accumulation of CD8+ T cells in Kras mutant lung tumor tissues (**Figure 3A**), a significant reduction of G-MDSCs and M-MDSCs within tumors along with a significant increase in antigen-presenting cells (**Figure 3B**). In addition, the effector function of CD8+ T cells was also promoted upon Mito-BEZ treatment, as indicated by upregulated transcription factor T-bet expression (**Figure 3C**), which is known to control the generation of CD8+ cytotoxic effector cells (25). Increased CD69 expression, a marker of early activation, was also observed in CD8 T cells (**Figure 3C**). Interestingly, the activation was only observed in CD8+ T cells but not in CD4+ T cells (**Figures 3C, D**). Mitochondrial complex III plays a pivotal role in maintaining the suppressive function of Tregs (26), to investigate if Mito-BEZ could directly affect Tregs’ suppressive function, *in vitro* T cell activation assay was conducted. CD4+ T cells isolated from the triple reporter SMARTA mice, which was generated by crossing SMARTA mice that express TCR specific for LCMV GP61-80 epitope, with interferon-gamma reporter GREAT mice and Foxp3^EGFP^ reporter mice, which resulted in the CD4+ T cells specifically activated by GP61-80 peptide, meanwhile express GFP or YFP to indicate the FOXp3 and IFNγ level. The CD4+ T cells were isolated from this triple reporter mice, activated, and cultured *in vitro* supplied with TGFβ (5 ng/mL) and IL-2 (100 U/mL), with or without BEZ and Mito-BEZ at varying concentrations for six days, flow cytometry analysis demonstrates that Mito-BEZ inhibited Foxp3+ Treg differentiation and/or survival and promoted Teff cell IFNγ production in a dose-dependent manner (**Figure 3E**). In contrast, BEZ did not appreciably inhibit Treg differentiation. Mito-BEZ potently inhibited mitochondrial complex I- and complex III-driven oxygen consumption. Parental compound BEZ only inhibited oxygen consumption by complex I- but not complex III-driven oxygen consumption. Thus, it is plausible that inhibition of Treg and stimulation of Teff response by Mito-BEZ are mediated by their increased potency to target mitochondrial complex III.

### Mito-BEZ distinctly modulates mitochondria in activated CD8+ T cells and cancer cells

**Figure 4:**
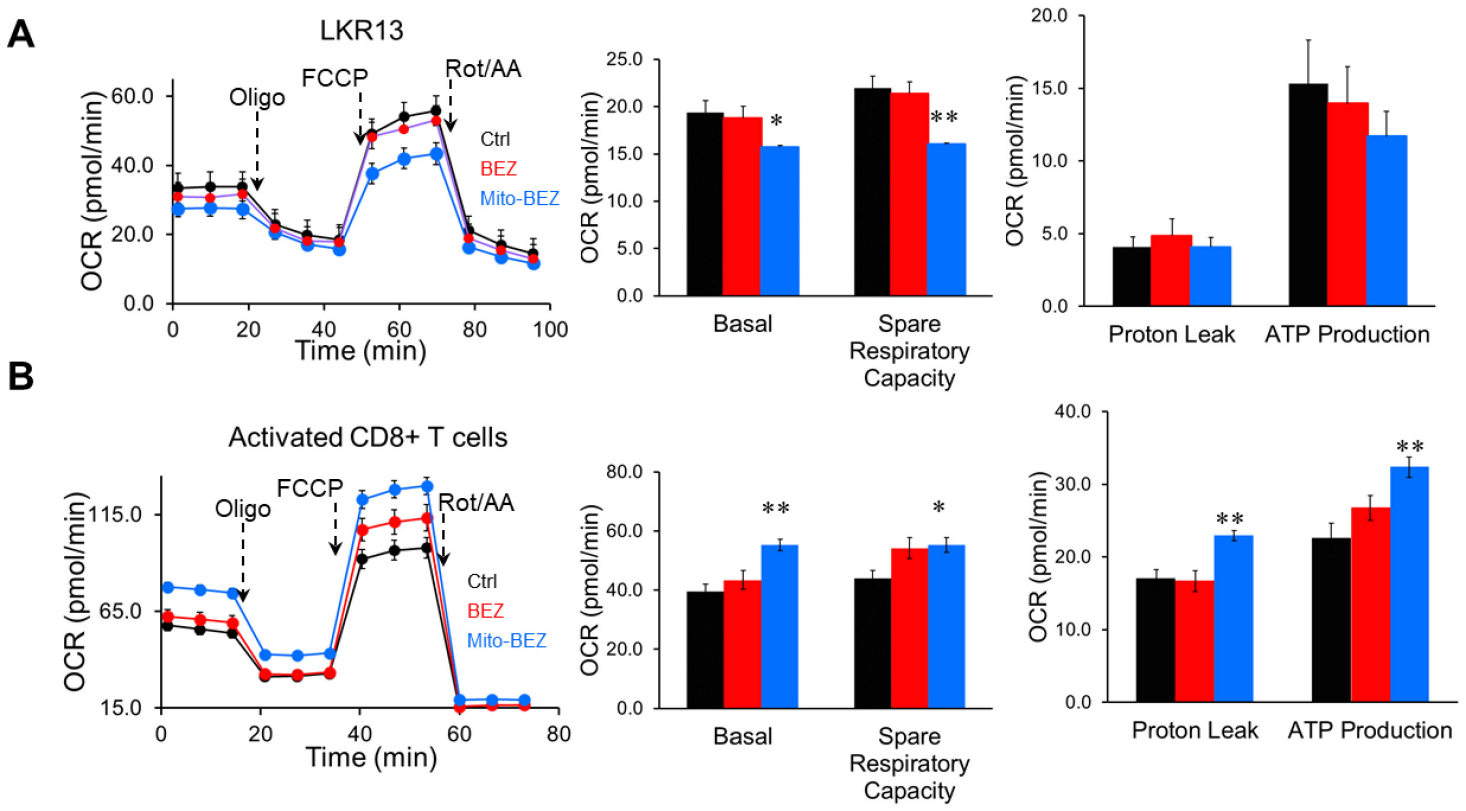
Mito-BEZ promotes mitochondrial function in activated CD8+ T cells. OCR from metabolic flux analyses in cancer cells (LKR-13, **A**) versus activated CD8+ T cells **(B)**. FCCP, carbonyl cyanide-4-(trifluoromethoxy)phenylhydrazone. *p ≤ 0.05, **p ≤ 0.01, ***p ≤ 0.001.

To delineate the possible mechanisms that led to enhanced CD8 T cell function, we compared the mitochondria function in cancer cells versus activated CD8+ T cells using Mito Stress assay, which measures the basal respiration, ATP-linked respiration, and maximal capacities, etc. in live cells in real-time. Metabolic flux analyses revealed that Mito-BEZ suppresses OXPHOS in cancer cells (**Figure 4A**), but dramatically upregulates oxidative metabolism and spare respiratory capacity (SRC) in CD8^+^ T cells as defined by their ability to increase OXPHOS above baseline (**Figure 4B**). Enhanced OXPHOS in T cells is consistent with metabolic characteristics of memory T cells and increased T cell function (27).

### Mito-BEZ prevents tumorigenesis in syngeneic mouse models

We compared the efficacy of Mito-BEZ and BEZ in lung tumor growth using a syngraft mouse model of lung cancer. At 8 weeks of age, LLC cells were injected subcutaneously. After 1 week, mice were treated by oral gavage 5 days per week with Mito-BEZ (3 μmol/kg) or BEZ (15 μmol/kg). BEZ was not effective at the dose (15 µmol/kg). In contrast, Mito-BEZ significantly decreased tumor progression at the dose of 15 µmol/kg (**Supplementary Figure S4**).

**Figure 5:**
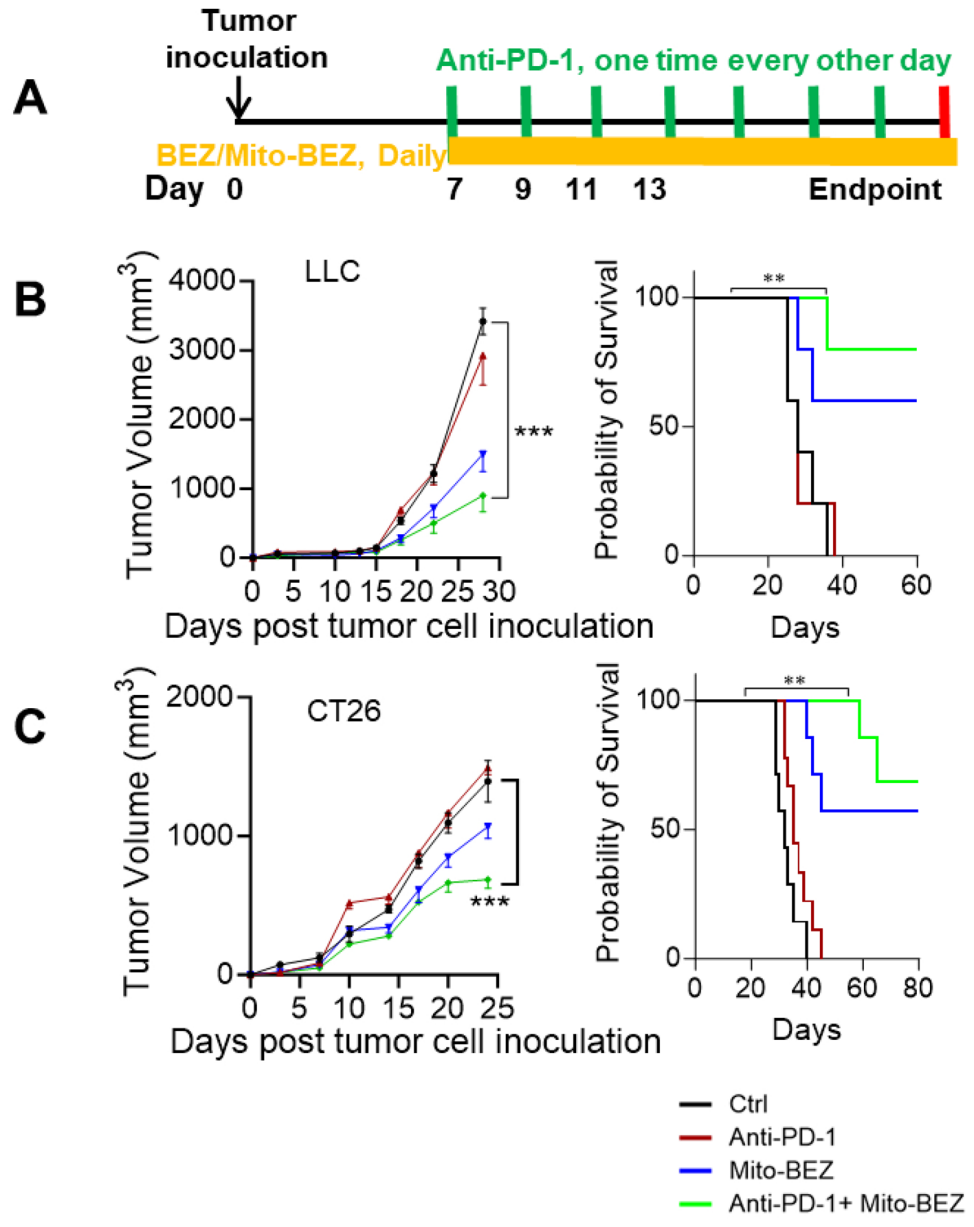
Mito-BEZ has a tumor-preventive effect in mice. **(A)** Experimental Design. **(B, C)** Efficacy and survival curve of BEZ in combination with anti-PD-1 monoclonal antibodies. n=5 mice per group, ***P < 0.001.

Since Mito-BEZ demonstrated significantly enhanced tumor-infiltrating T cells, especially activated CD8+ T cells through elevated mitochondria function, we speculated that mito-BEZ would facilitate better responses to immune checkpoint blockades, therefore, we tested this theory in two well-known immune checkpoint inhibitor (ICI)-resistant syngeneic mouse tumor models. We implanted mice with syngeneic lung cancer LLC or colon cancer CT26 cells subcutaneously and treated them with Mito-BEZ by oral gavage daily for four weeks. The tumors of vehicle-treated mice grew progressively. All treatments did not have a significant effect on body weight in both models (**Supplementary Figure S5**). We combined Mito-BEZ with anti-PD-1 checkpoint immunotherapy (**Figure 5A**). We observed that Mito-BEZ alone significantly decreased tumor load, while anti-PD-1 did not decrease tumor load in these two models by itself. However, a combination of anti-PD-1 antibody with Mito-BEZ resensitized these tumors to ICIs and demonstrated superior antitumor efficacy (**Figures 5B, C**). Furthermore, a notable survival advantage was seen in mice given Mito-BEZ plus anti-PD-1 antibodies compared with the control groups (**Figures 5B, C**).

## Discussion

Targeting mitochondrial metabolism presents a relatively new concept in cancer prevention and therapy (28, 29). Lipophilic and cationic TPP+-conjugated bioactive agents readily diffuse across cell membranes, lack the toxicity associated with traditional mitochondrial OXPHOS inhibitors, and cumulatively have a high therapeutic index (1–3). In previous studies, we established that TPP^+^-containing mitochondria-targeted drugs are driven into cancer cell mitochondria by increased negative mitochondrial membrane potential. These TPP+ drugs potently and selectively inhibit OXPHOS and induce redox signaling in cancer cells (16). Cationic TPP^+^-conjugated bioactive agents have a high therapeutic index, and lack the toxicity associated with traditional mitochondrial OXPHOS inhibitors.

BEZ has been repurposed in cancer therapy, it can enhance proliferation of CTLs and prime CTLs to generate more effective T cells by modulating mitochondrial biogenesis in T cells (30). However, the potency of BEZ in tumor cells is modest even when used at relatively higher doses (e.g. 200mg/KG) (7, 31). We were therefore motivated by the hypothesis that targeting BEZ to mitochondria with TPP+ cationic approach may enhance its antitumor immune responses. Indeed, in our current study, with the modification of adding TPP+ to BEZ, Mito-BEZ has shown much enhanced efficacy compared to its parental compound BEZ in anticancer efficacy. Mito-BEZ is over 400-fold more potent in inhibiting mitochondrial oxygen consumption than BEZ, which results in a significantly enhanced tumor inhibition in LUAD cells.

Increasing evidence suggests that alterations in metabolism can have substantial immune effects. BEZ has a synergistic antitumor effect with PD-1 blockade in mice (30, 32, 33), and data from a phase 1 clinical trial indicates that BEZ indeed promotes T cell function through the activation of mitochondria in T cells (33). In the current study, tumor extrinsic mechanisms impacted by Mito-BEZ were also evaluated. Mito-BEZ exerts immunomodulatory effects on immune cells in the TME through altered mitochondrial bioenergetics and redox metabolism primarily on CD8+ T cells. We found that Mito-BEZ induces a stronger immune response than BEZ in the TME via mitochondrial mechanisms. Mito-BEZ promoted mitochondrial function with much higher capacity compared to BEZ, and upregulated ATP production and glycolysis, both are essential for the effector CTLs. These positive modulation within TME certainly orchestra well with PD-1 blockade treatment, resulting in significantly enhanced anti-cancer efficacy in PD-1 resistant tumor models, and prolonged overall survival.

Overall, Mito-BEZ is a novel, potent chemopreventive agent of LUAD that not only inhibits cancer cell metabolism but also boosts antitumor immunity in the TME.

## Data Availability

The original contributions presented in the study are included in the article/**Supplementary Material**. Further inquiries can be directed to the corresponding author.
