# Supplementary figures and images for: Bezafibrate-driven mitochondrial targeting enhances antitumor immunity and prevents lung cancer via CD8+ T cell infiltration and MDSC reduction
Source: Front Immunol. 2025 Apr 15;16:1539808. doi: 10.3389/fimmu.2025.1539808 (PMC12037589; doi:10.3389/fimmu.2025.1539808)

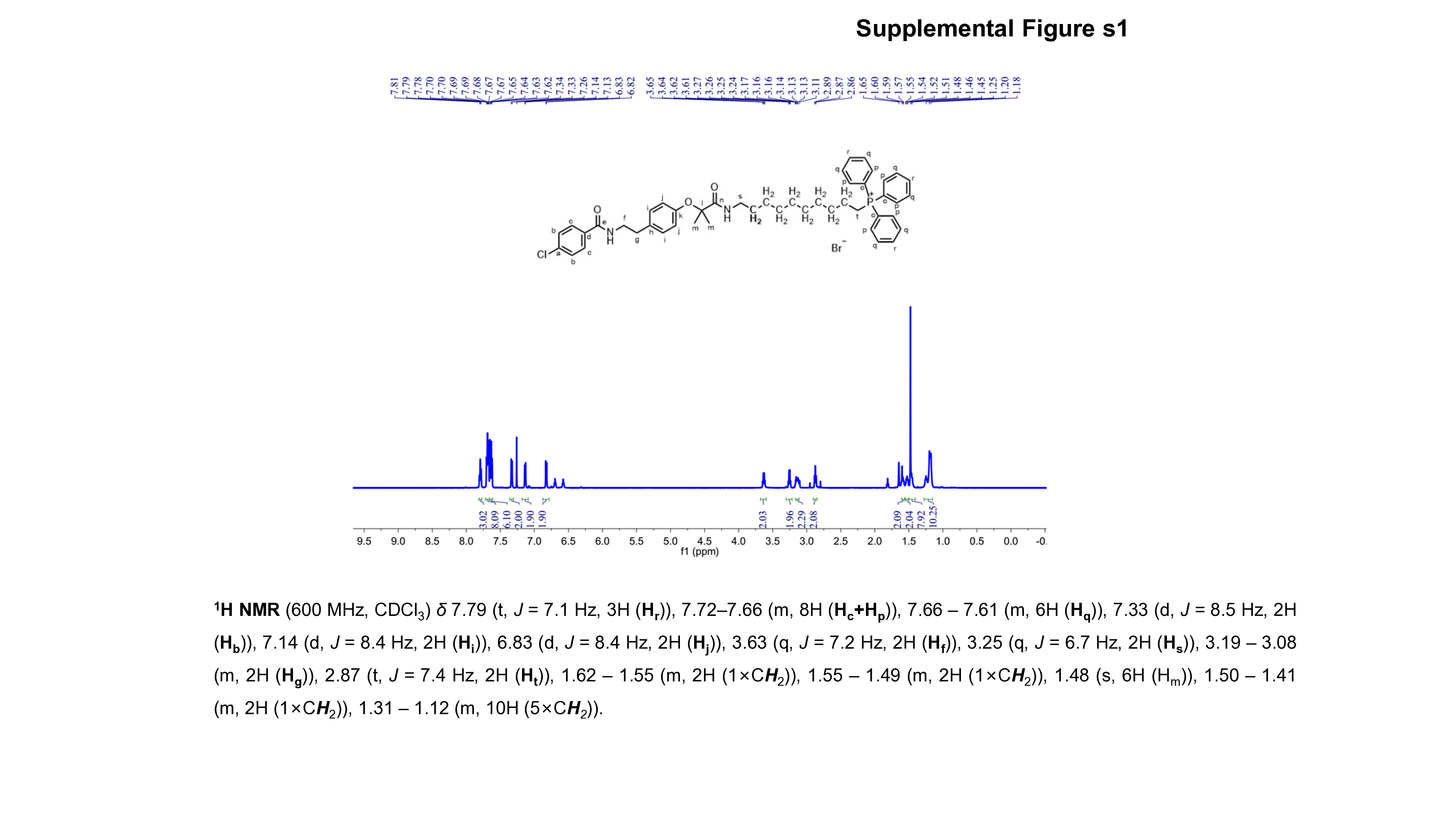

Supplement: Supplementary file 1 [file Image1.jpg]

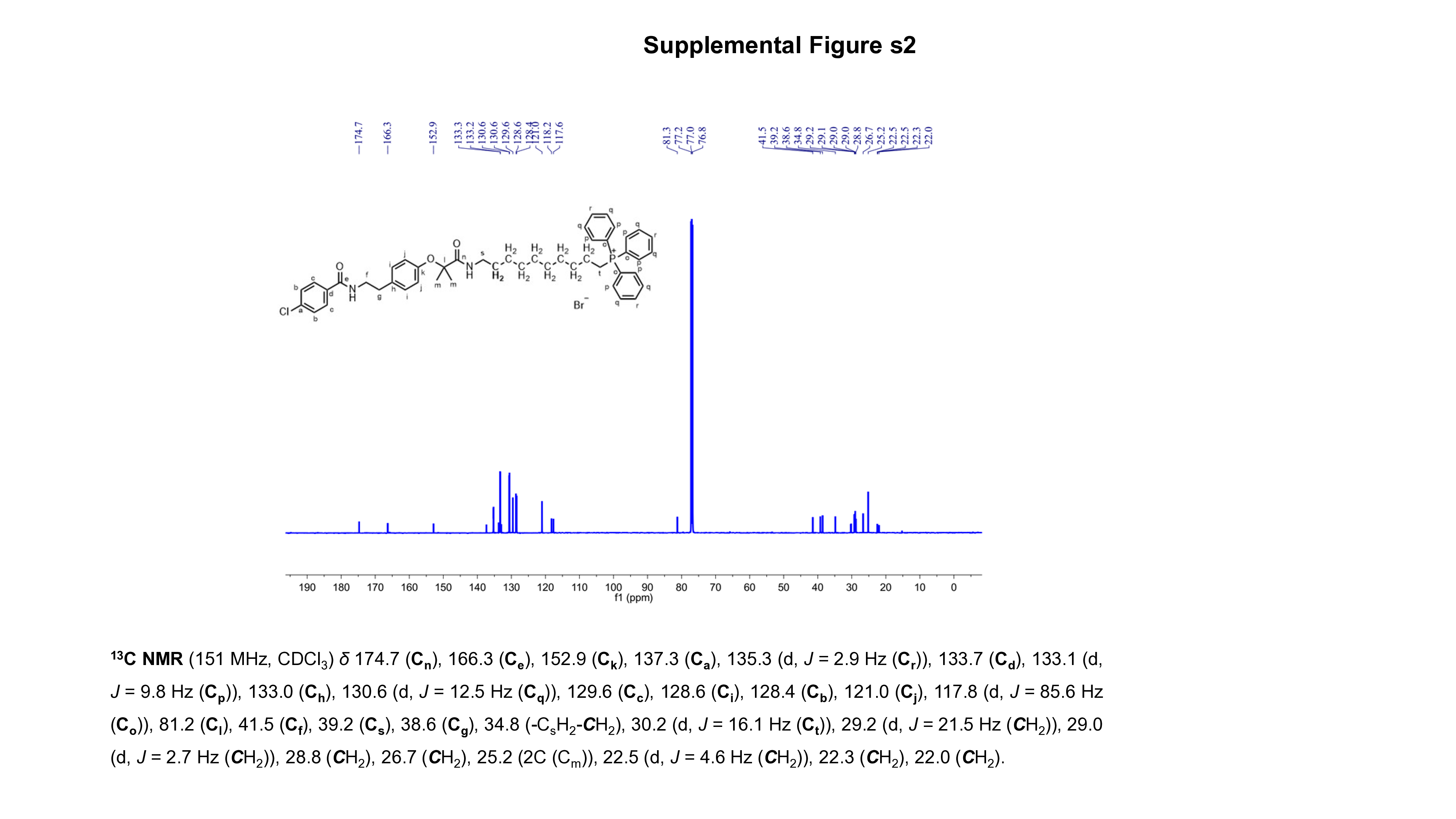

Supplement: Supplementary file 2 [file Image2.jpg]

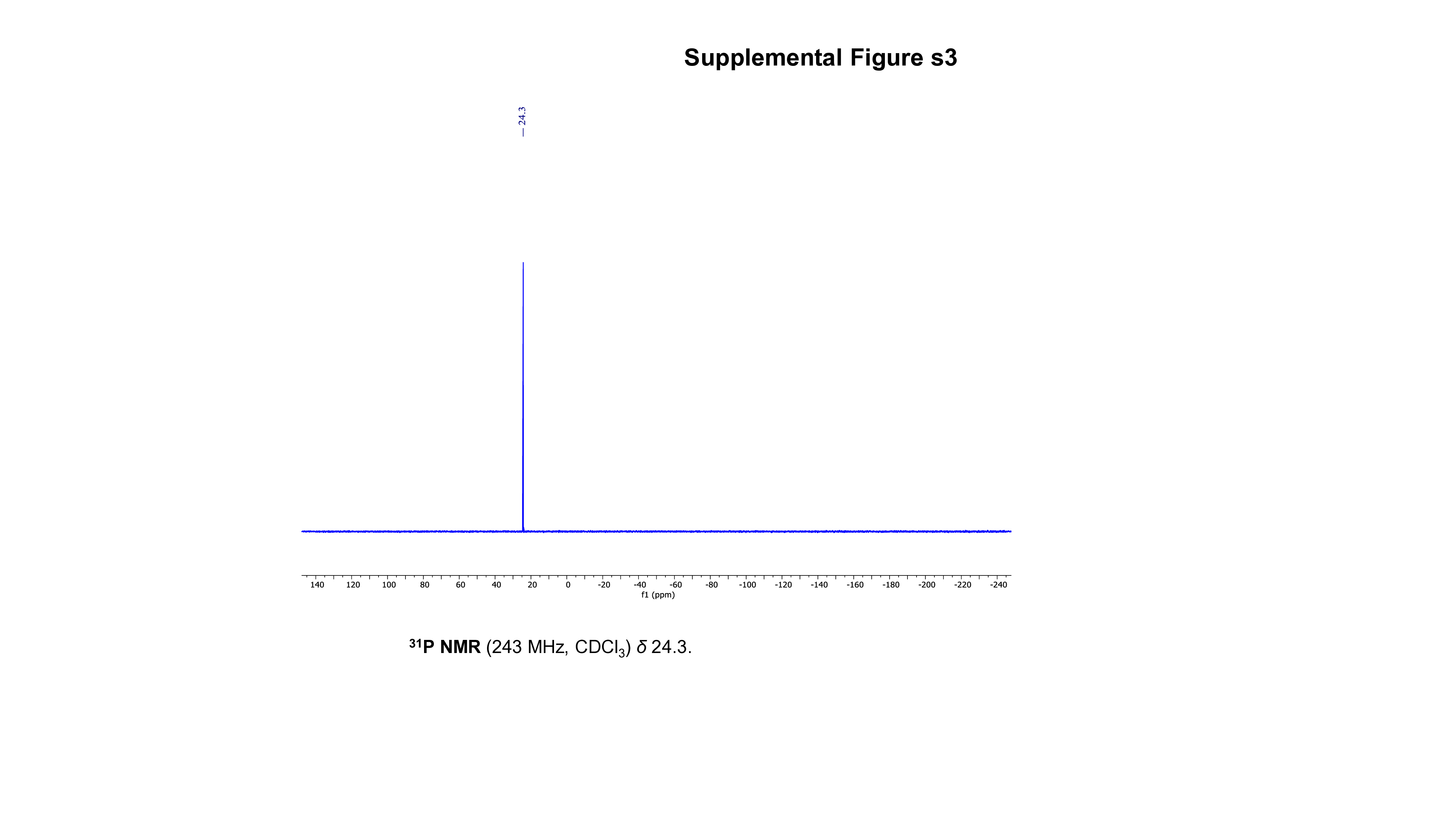

Supplement: Supplementary file 3 [file Image3.jpg]

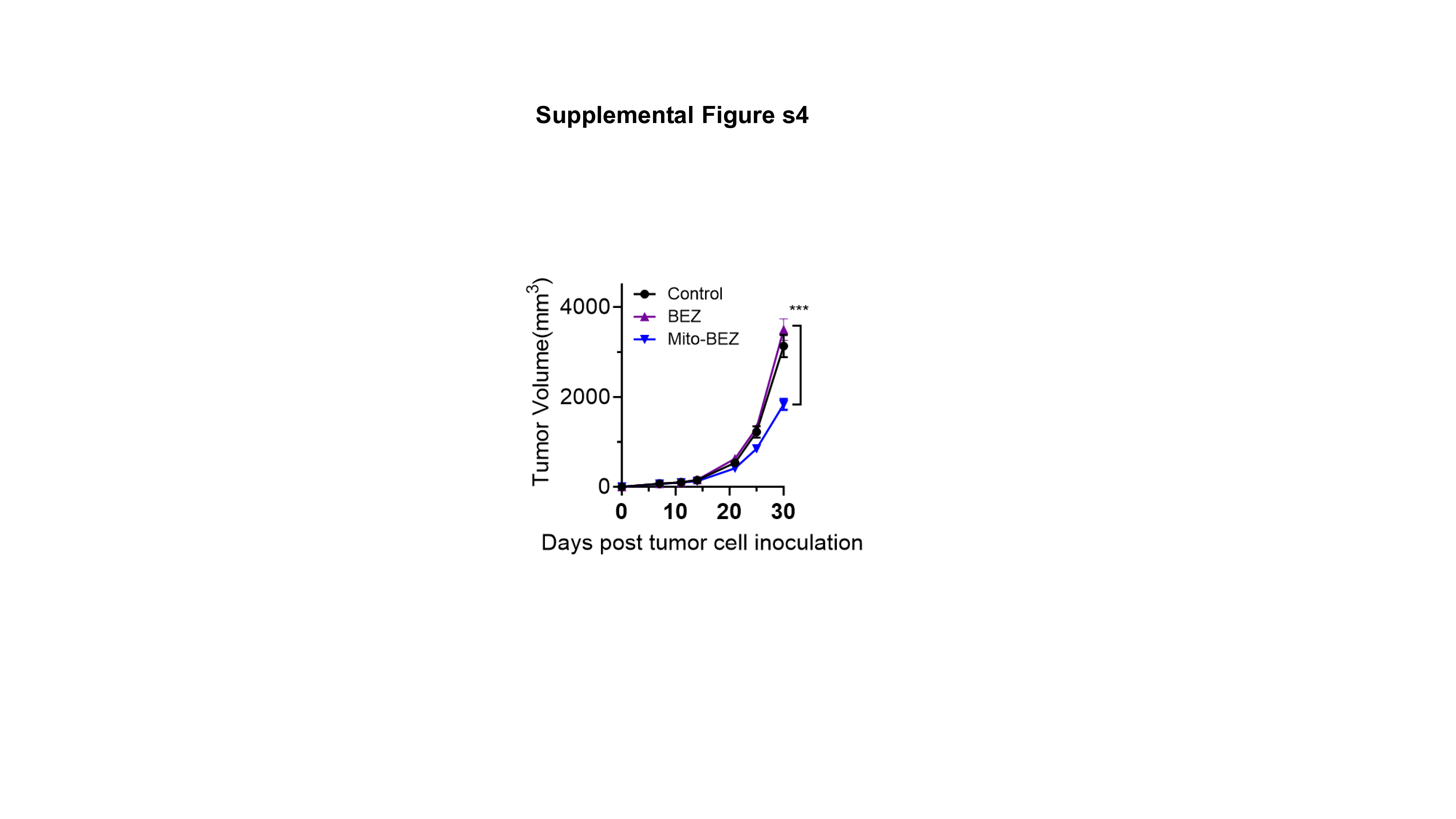

Supplement: Supplementary file 4 [file Image4.jpg]

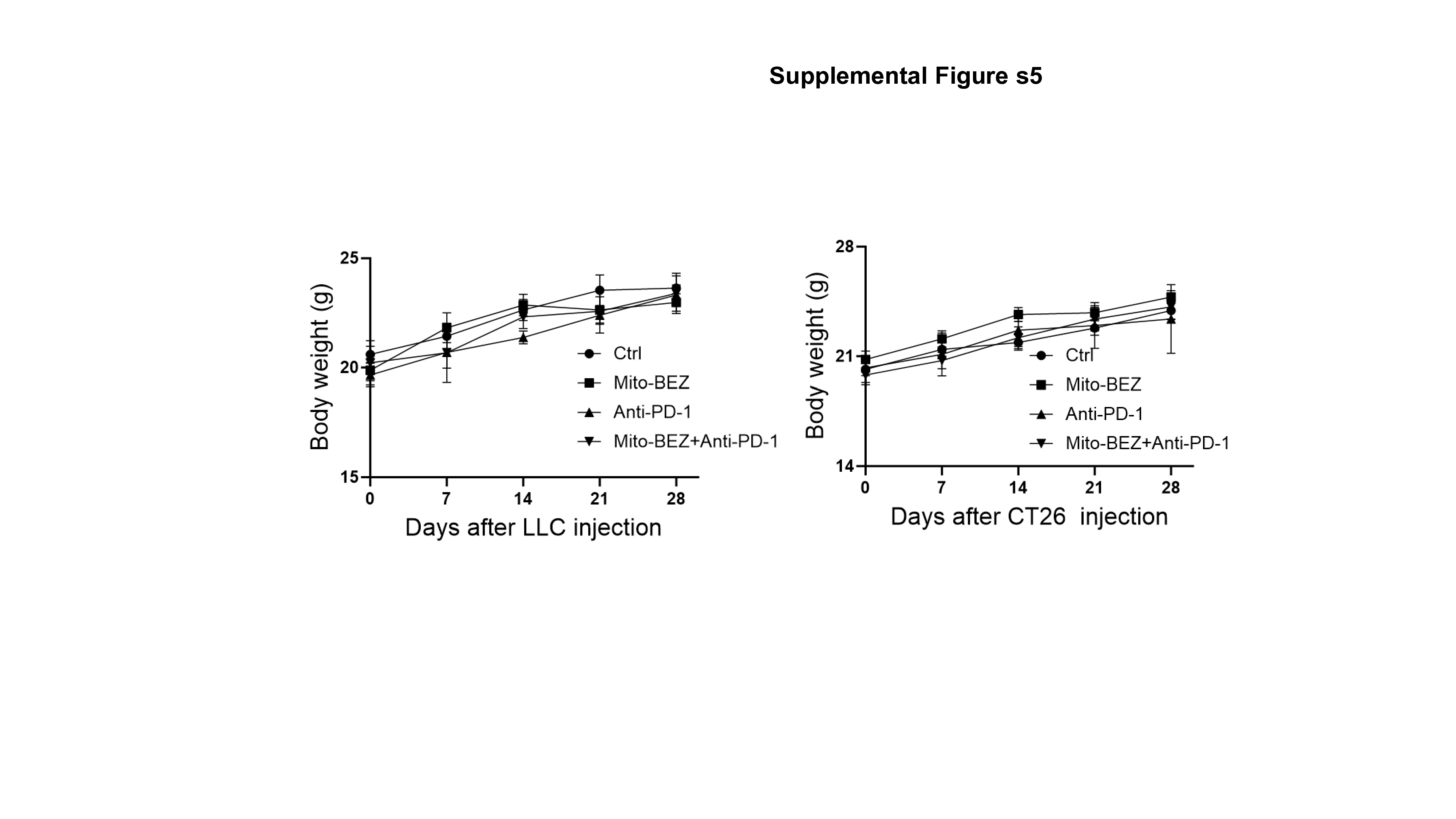

Supplement: Supplementary file 5 [file Image5.jpg]
